# Factors Associated with Anemia among Children Aged 6–23 Months Attending Growth Monitoring at Tsitsika Health Center, Wag-Himra Zone, Northeast Ethiopia

**DOI:** 10.1155/2015/928632

**Published:** 2015-05-27

**Authors:** Haile Woldie, Yigzaw Kebede, Amare Tariku

**Affiliations:** ^1^Department of Human Nutrition, Institute of Public Health, College of Medicine and Health Science, University of Gondar, Gondar, Ethiopia; ^2^Department of Epidemiology and Biostatistics, Institute of Public Health, College of Medicine and Health Science, University of Gondar, Gondar, Ethiopia

## Abstract

*Background*. Globally, about 47.4% of children under five are suffering from anemia. In Ethiopia, 60.9% of children under two years are suffering from anemia. Anemia during infancy and young childhood period is associated with poor health and impaired cognitive development, leading to reduced academic achievement and earnings potential in their adulthood life. However, there is scarcity of information showing the magnitude of iron deficiency anemia among young children in Ethiopia. Therefore, this study aimed at assessing prevalence and associated factors of iron deficiency anemia among children under two (6–23 months). *Methods*. Institution based cross-sectional study was carried out from March to May, 2014, at Tsitsika Health Center in Wag-Himra Zone, Northeast Ethiopia. Systematic random sampling technique was employed. Automated hemoglobin machine was used to determine the hemoglobin level. Socioeconomic and demographic data were collected by using a pretested and structured questionnaire. Binary logistic regression analysis was used to identify associated factors and odds ratio with 95% CI was computed to assess the strength of association. *Results*. Total of 347 children participated in this study. The overall prevalence of anemia was 66.6%. In multivariate logistic regression analysis, male sex (AOR = 3.1 (95% CI: 1.60–5.81)), 9–11 months of age (AOR = 9.6 (95% CI: 3.61–25.47)), poor dietary diversity (AOR = 3.2 (95% CI: 1.35–7.38)), stunting (AOR = 2.7 (95% CI: 1.20–6.05)), diarrhea (AOR = 4.9 (1.63–14.59)), no formal education (AOR = 2.6 (95% CI: 1.26–5.27)), early initiation of complementary food (AOR = 11.1 (95% CI: 4.08–30.31)), and lowest wealth quintile (AOR = 3.0 (95% CI: 1.01–8.88)) were significantly associated with anemia. *Conclusion*. The overall prevalence of anemia among children who aged 6–23 months has sever public health importance in the study area. Integrated efforts need to be prioritized to improve health as well as appropriate infant and young child feeding practice among children under.

## 1. Background

Currently, micronutrient deficiencies are coming to be the most prevalent nutritional deficiencies causing serious developmental problems in the world [1]. Anemia is one of micronutrient deficiencies which have serious public health significance in the world. It is second leading nutritional cause of disability [2–4]. Anemia is an important outcome indicator of poor nutrition and health with its major consequences on socioeconomic development of a population [5]. Anemia can occur at any time and at all stages of the life cycle [3] but young children and pregnant women are the most at risk segment of the community [1].

Globally, about 47.4% of children under five are suffering from anemia [6]. In developing countries, it affects 46–66% of children aged under five years [7]. Africa and Asia are found with sever public health importance of anemia [8]. About 67.6% of children under five in Africa are suffering from anemia while they are 65.5% in Southeast Asia [6]. The highest overall prevalence of anemia in children aged under 5 years is recorded in the Western and Central African Region as 75% [9]. According to the Ethiopian 2011 DHS report, the overall prevalence of anemia among children under two years (6–23 months) was 60.9% [10].

Among children, iron deficiency anemia is consequence of complex interaction of several factors. As different studies claimed that iron deficiency anemia is significantly associated with low birth weight [11], sex [11–14], age [11, 12, 14–16], rural residence, infectious disease (malaria, tuberculosis, intestinal parasitic infestation, and HIV/AIDS) [15, 17], undernutrition (stunting, wasting, and underweight) [13, 15, 18], poor socioeconomic status [12, 14, 16, 18], household food insecurity [12], duration of lactation [18] and poor dietary iron intake [18], poor maternal educational status [11, 14, 18], and maternal anemia [12].

Anemia during childhood period is strongly associated with poor health and physical development [3, 19], mild and moderate mental retardation [3, 20], and poor motor development and control [16, 21] leading to reduced academic achievement and work capacity thereby reducing earning potential and damaging national economic growth in the future [19]. Iron deficiency anemia also increases risk of mortality and morbidity from infectious disease [3, 11, 22].

Still anemia with its devastating implication has public health importance among children under five in Africa [23]. In Ethiopia, as part of other African countries, burden of anemia also proves public health significance among children [10]. However, there is scarcity of information showing the burden of anemia and its risk factor among children under two. Therefore, this study was aimed to assess prevalence of anemia and its associated factors among children aged 6–23 months in Wag-Himra zone, Northeast Ethiopia.

## 2. Methods

### 2.1. Study Area

This study was conducted at Tsitsika Health Center, Ziquala Woreda, Amhara regional state, Northeast Ethiopia. Tsitsika (Woreda capital of Ziquala Woreda) is 730 km far from Addis Ababa, the capital city of Ethiopia, and founded at 900 meters above sea level. According to the regional finance and economic development projection, the total population of the study area for the year 2014 was 49671. The total numbers of children aged under five and under two years were 6987 and 2473, respectively. Residents of this Woreda largely depend on subsistence farming and producing cereals as main agricultural product. Since 2008, the study area has been implementing community based nutrition intervention program (growth monitoring, nutritional screening, vitamin-A supplementation, outpatient therapeutic feeding program, etc.). At the time of the study, Ziquala Woreda has six health centers and 15 health posts. Growth monitoring targeting children under two years has been carried out by using Weight-for-Age anthropometric index as routine procedure in all health institutions of current study area.

### 2.2. Study Population and Sampling Techniques

All children aged 6–23 months with mothers/caretakers attending growth monitoring clinic during the study period were included in the study. Those children who did not register from the Health Center logbook for their health status were excluded from the study. Systematic random sampling technique was employed to recruit a total of 347 children. The total sample size was determined by using the formula to estimate a single population proportion with the following assumptions: population proportion (*P*), that is, 60.91% taken from Ethiopian 2011 DHS report as prevalence of anemia among children aged 6–23 months, 95% confidence level, and 5% margin of error. Then, the final sample size 366 was obtained. The sampling interval was calculated from the 2013 growth monitoring registration book of the Health Center. The number of children aged 6–23 months and participated for growth monitoring during calendar year of March to May/2013 was taken as 763 from the logbook. Children who have no record regarding their morbidity status on the registration book were not included in the study. Sampling interval was determined by 763/366 = 2.01 ≈ 2; then, lottery method was employed to select starting or first sample in the study. The procedure continued until the required sample size was obtained.

### 2.3. Data Collection Tools and Procedures

Socioeconomic and demographic characteristics of the family and child, feeding practice, health care utilization, and child morbidity status (within two weeks before data collection) were collected by using a pretested and structured questionnaire through interviewing of the mother/caretakers of the child. Hemoglobin level of the child was measured from capillary blood and one drop of capillary blood was carefully collected from the middle finger of the child by finger prick. Strict aseptic technique and a separate lancet for each child were employed. Automated hemoglobin machine made from Germany with model kx-21 and serial number b-0839Model was used to determine the hemoglobin concentration and the result was expressed in g/dL and the presence and severity of anemia were determined according to age based criteria of WHO cut-off point. For children aged 6–24 months who have hemoglobin level >11 g/dL, they were considered as nonanemic, 10.0–10.9 g/dL as mildly anemic, 7–9.9 g/dL moderately anemic, and <7 g/dL as severely anemic [24].

Nutritional status of the child was assessed by taking anthropometric body measurement of the child. Length of a child was measured in a recumbent position to the nearest 0.1 cm by using a board with an upright wooden base and a movable headpiece, on a flat surface. Weight measurement of a child was taken by a Salter scale (model-2356S) with the calibration of 100 g unit. It is designed and manufactured under the authority and recognition of the United Nations Children's Fund (UNICEF). The scale was adjusted to zero before weighing every child and measurement was recorded to the nearest 0.1 kg. Each measurement was repeated and the mean value was calculated and recorded on the questionnaire. All children were without any shoes during the measurement.

Age of child was determined by two methods: for 81 children aged between 12–23 months, their birth date was extracted from the immunization card; while for 266 children without immunization card and who aged under 12 months, their age was determined by using information given by the mother/female caretaker of the child. The three standard indices (Length-for-Age, Weight-for-Length, and Weight-for-Age) were analyzed by ANTHRO software and used to determine the nutritional status of children. Each of the three measurements was expressed in standard deviation units of* Z*-score from the median of WHO-2006, standard population [25]. Children with a measurement <−2 of* Z*-score were determined as stunted for Length-for-Age, wasted for Weight-for-Length, and underweight for Weight-for-Age. Information related to morbidity status of the child (intestinal parasite, diarrhea, malaria, and upper respiratory tract infection) before two weeks was captured by looking up the health centre logbook.

### 2.4. Measurement of Dietary Diversity Score

Dietary diversity scores of a child were determined by using WHO and “indicators for assessing infant and young child feeding practices” minimum dietary diversity for children age 6–23 months and by employing 24 hrs recall method. Mothers or female care takers were asked to report all food items and beverages given to the child during the previous day of the survey. Then, all food items and beverages consumed by the child were categorized into seven food groups as (1) grains, roots, and tubers, (2) legumes and nuts, (3) dairy products, (4) flesh foods, (5) eggs, (6) vitamin-A rich fruits and vegetables, and (7) other fruits and vegetables [26]. Using dietary diversity score 4 (minimum dietary diversity score) as cut-off point, a child was defined as having “poor dietary diversity” if he/she consumed less than 4 food groups while having “good dietary diversity” if he/she had 4 or more food groups.

### 2.5. Determination of Wealth Index

The wealth index was used in the study and constructed from the data collected in the household questionnaire. The standardized tool for measurement of wealth index was adopted from Ethiopian DHS-2011 [10]. This index consist of seven selected household asset data, that is, availability of electric city, television, refrigerator, mobile telephone, nonmobile telephone, a bed with cotton/sponge/spring mattress, and electric mittade (local name for electric stove or oven), and via a principal components analysis. The wealth index was divided into five categories (lowest, second, middle, fourth, and highest).

### 2.6. Data Quality Control

Two-day intensive training was given for data collectors and supervisors regarding study objective, interview techniques, anthropometric measurements, and ethical issues during data collection. Pretest without hemoglobin level determination was done among 5% of the total sample size in the nearest health postproviding growth monitoring service before three days of the actual data collection in order to sort out language barriers and contextual difference on the structured questionnaires. Questionnaire was checked daily for accuracy, consistency, and completeness by supervisor. Furthermore, the supervisors and the principal investigator give feedback and correction regarding the collected data on daily basis to the data collectors.

### 2.7. Data Processing and Analysis

Data was cleaned, coded, and entered using EPI-INFO version 3.5.3 and exported to SPSS version 16 for analysis. Bivariate analysis was done to see the association of each independent variable with the outcome variable (anemia status). Those independent variables having *P* value less than 0.2 in the bivariate analysis were entered into the multivariate analysis to determine the effect of each explanatory variable on outcome variable and to control the possible effect of confounders. Odds ratio with 95% confidence level was used to determine the strength of association. In the multivariate analysis, independent variables with *P* value ≤ 0.05 were considered as significant.

### 2.8. Ethical Consideration

Ethical approval was obtained from Institutional Review Board of University of Gondar. Each mother/caretaker was informed about the objective of the study and written informed consent was secured before questionnaire administered. A child with a confirmed anemia was referred to the concerned body in the Health Center.

## 3. Results

### 3.1. Socioeconomic and Demographic Characteristics of a Child and Family

A total of 347 children aged 6–23 months with their mothers/caretakers were included in the study giving response rate of 97%. Fourteen percent of mothers/caretakers of children had no formal education. Eighty percent of children were living with both parents and 55.9% had one sibling aged under five years. Nineteen percent of families were at the lowest level of wealth quintile range and 10.4% were at the highest one (Table 1).

### 3.2. Feeding Practice and Nutritional Status of Children

Only one child was found without history of ever breast feed and 88.2% of children were found with history of current breast feeding status during the interview. About 20.5% of children had early introduction of complementary foods while 25.1% were found with history of cow's milk consumption before 12 months of their age. Eighty five percent of children had poor dietary diversity scores. Nearly 24% of children were stunted (Table 2).

### 3.3. Morbidity and Health Care Related Characteristics of the Child

About 59.4% of children were born at home. Regarding the morbidity status, 14.4% were with malaria infection and 14.7% of children had diarrhea in the last two weeks (Table 3).

### 3.4. Prevalence of Anemia among Children Aged 6–23 Months

The overall prevalence of anemia was 66.6%. Burden was higher among males with the magnitude of 55.4%. Among the four age groups, the highest prevalence was recorded in the age group of 9–11 months (79.6%), followed by 6–8 months (69.2%).

### 3.5. Factors Associated with Anemia among Children Aged 6–23 Months

The result of both bi- and multivariate analyses revealed that sex of the child, age, history of diarrhea before two weeks, maternal educational status, dietary diversity, introduction of complementary foods, stunting, and household wealth quintile were significantly associated with the anemia (Figure 2 and Table 4).

## 4. Discussion

**Figure 1:**
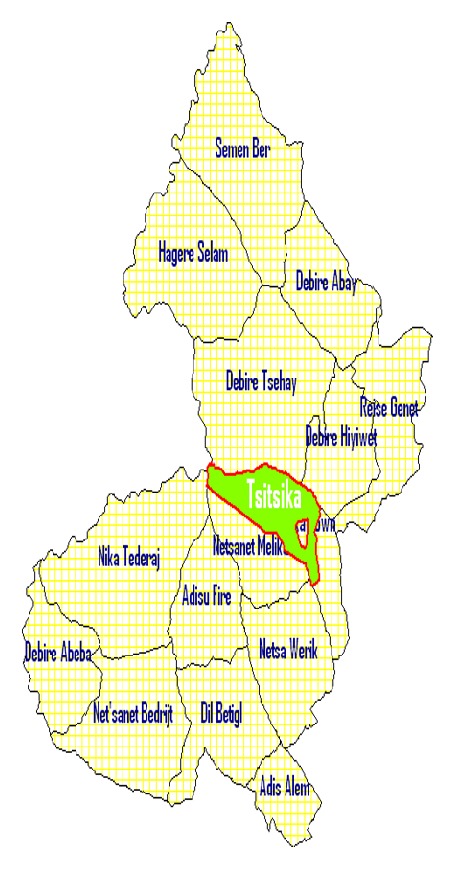
Map of Ziquala Woreda (study area), Northeast Ethiopia.

**Figure 2:**
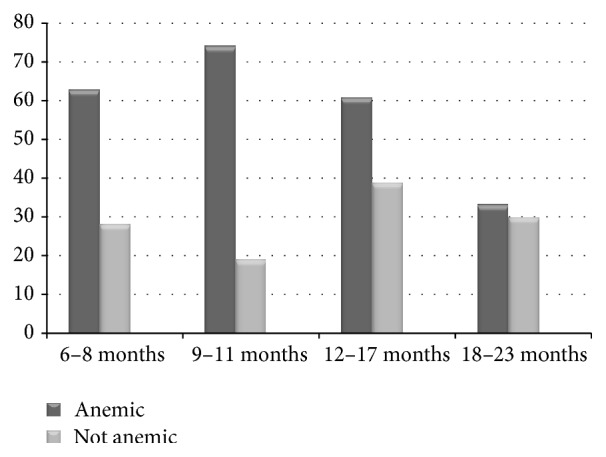
Number of children (6–23 months) with anemia by age category.

The result of this study revealed that 66.6% of children were anemic (95% CI: 0.619–0.713). The result is slightly higher than 2011 Ethiopian DHS report, 60.9% [10], and Bangladesh, 60% [27]. But the finding is lower than study report in Nepal, 69% [28], and Ghana, 84.3% [29]. This could be because, in developing countries, complementary foods for children are mostly porridges made of locally available staple cereals [30]. Cereals are known to be rich in phytates, which are nutrients causing poor bioavailability of iron. Similarly, staple food in the study area is cereal based given that children in the current study area share the same risk with other developing countries. Approximately 36% of children were with history of early and late introduction of complementary foods. Both carries risks contributing to persistent young child malnutrition [30]. Both practices are well known to cause anemia among young children. The current study area (Figure 1) is one of chronical foods in secured area in the region. This household food insecurity might hinder child from obtaining adequate and appropriate complementary food due to poor household food purchasing power.

Male children were 3.1 times more likely to be anemic as compared to females (AOR = 3.1 (95% CI: 1.60–5.81)). This finding is similar with study reports in Ghana [29] and Bangladesh [27]. Other studies conducted in Tanzania [31] and Brazil [32] found that sex difference did not show association with anemia. The possible explanation for this discrepancy could be due to sate of rapid growth of male children in the first months of life which increases their micronutrient requirement including iron [33]. If this physiological state is not compensated with appropriate and iron rich complementary foods at this critical stage, risk of iron deficiency anemia will be higher among male children as compared to their counterpart.

Children in the age group of 6–8, 9–11, and 12–17 months were 3.5 times (AOR = 3.5 (95% CI: 1.46–8.26)), 9.6 times (AOR = 9.6 (95% CI: 3.61–25.47)), and 2.9 times (AOR = 2.9 (95% CI: 1.23–6.75)) more likely to be anemic than children in the age range of 18–23 months, respectively. This could be because prenatal iron store depletion is highest starting at six months of age [34]. In addition, it may be due to poor maternal iron reserve during pregnancy. It is known that the anemic pregnant mothers are more likely to give birth of child with poor iron stores [12].

Children with early (<6 months) and late (≥9 months) introduction of complementary foods were 11.1 times (AOR = 11.1 (95% CI: 4.08–30.31)) and 4.3 times (AOR = 4.3 (95% CI: 1.78–10.18)) more likely to be anemic than children with timely initiation of complementary food, respectively. It is evident that most digestive enzymes are inadequate until the first six months of age [35] and introducing liquid or solid food during this time causes interference with the absorption of iron in the breast milk [36]. Early exposure of infants (before six months of age) to microbial pathogens due to complementary foods increases the risk of infection for diarrheal disease, thereby malabsorption [37]. Breast milk has minimal iron to fulfill nutritional requirement of growing infant [38], given that providing breast milk alone coupled with rapid iron depletion beyond six months also increases risk of anemia for younger infant.

Those children with poor dietary diversity score were near to three times more likely to be anemic than children with good dietary diversity scores (AOR = 3.2 (95% CI: 1.35–7.38)). Cereal based monotonous diets (undiversified diet) are known to cause micronutrient deficiency including anemia [39]. It is also evidenced that dietary diversity is proxy indicator for micronutrient adequacy of diet [40].

Children with history of diarrhea before two weeks of the study were 4.9 times more likely to be anemic than children without diarrhea (AOR = 4.9 (1.63–14.59)). This finding is consistent with study reports in Indonesia [41, 42] and Brazil [32]. This could mainly operate through loss of appetite and malabsorption from diarrhea which in turn increases likelihood of developing anemia.

Stunted children were 2.7 times more likely to be anemic than their counterpart (AOR = 2.7 (95% CI: 1.20–6.05)). This finding is similar to studies conducted in Bangladesh [27], Brazil [43], and Burma [44]. This is could be because undernourished children are often anemic [43], low hemoglobin level has compromising effect of the linear growth [45], and coexisting of other micronutrient deficiencies and stunting may increase the development of anemia by a synergism association. But the current study cannot provide cause and effect relationship between stunting and iron deficiency anemia.

Children of mothers with no formal education were 2.6 times more likely to be anemic than children of mother with secondary and above education level (AOR = 2.6 (95% CI: 1.26–5.27)). This finding is similar to study conducted in Kenya [14], Ghana [39], and Bangladesh [28]. But a study conducted in Timor-Lest [46] reported that maternal educational status was inversely associated with their children's nutritional status. Children of mothers with secondary education had significantly lower mean hemoglobin concentration than mothers with primary and no education. Moreover, mothers' level of education may positively influence practices related to the health care and feeding practice of their children. Educated mothers are more conscious of their children's health and introducing scientifically proved feeding practices, which help to improve their children nutritional status [28]. It is also confirmed that, maternal education is strong predictor for nutritional outcomes of children [39].

Children from families of lowest wealth quintiles were three times more likely to be anemic than children from highest wealth quintile (AOR = 3.0 (95% CI: 1.01–8.88)). The finding of the current study is in line with studies conducted in Ghana [39], Brazil [33], and Bangladesh [28]. Poor household economic status might result in loss of power to purchase diversified and nutrient rich food and secure the household per capita food availability. Other studies also reveal that poor household economic status is associated with household food insecurity [22]. In turn, household food insecurity is strong determinant factor for undernutrition including iron deficiency anemia [12, 47].

Some limitations of the study should be noted and taken into consideration. Cross-sectional nature of this study did not reveal causal links between independent variables and iron deficiency anemia. Anemia status from the study group was determined by using the hemoglobin level but not by using the latest test indicators like serum ferritin.

## 5. Conclusion

Burden of anemia among children aged 6–23 months in the study area is higher and it has severe public health significance according to the WHO cut-off points. Stunting, mother with no formal education, lowest wealth quintiles, having diarrhea before two weeks, poor dietary diversity scores, early and late introduction of complementary foods, and sex and age of the child were significantly associated with anemia. Well integrated interventions to improve the health status and infant and young child feeding practices need to be prioritized to prevent deficiency of anemia targeting children aged under two years of age.

## Figures and Tables

**Table 1:** Socioeconomic and demographic characteristics of the family and children aged 6–23 months attending growth monitoring at Tsitsika Health Center, Northeast Ethiopia (*n* = 347).

Background characteristics	Frequencies	Percent (%)
Sex of the child		
Male	171	49.3
** **Female	176	50.7
Age of the child (in months)		
** **6–8 months	91	26.2
** **9–11 months	93	26.8
** **12–17 months	100	28.8
** **18–23 months	63	18.2
Respondent relation to the child		
** **Mother	335	96.5
** **Other	12	3.5
Marital status of the mother/caretaker		
** **Single	36	10.4
** **Married	306	88.2
** **Other^*^	5	1.4
Educational status of the mother/caretaker		
** **No formal education	140	40.3
** **Primary education	114	32.9
** **≥Secondary education	93	26.8
Employment status of the mother/caretaker		
** **Housewife	266	76.7
** **Civil servant	27	7.8
** **Farmer	27	7.8
** **Merchant	11	3.2
** **Other^**^	16	4.6
Father educational status		
** **No formal education	78	22.7
** **Primary education (1–8)	147	42.4
** **≥Secondary education and above	121	34.9
Father employment status		
** **Farmer	149	42.9
** **Civil servant	74	21.3
** **Merchant	59	17.0
** **Private employed	47	13.5
** **Other	18	5.2
Birth order of the child		
1st	65	18.7
2nd	74	21.3
3rd	69	19.9
4th and above	139	40.1
Living arrangement of the child		
** **Living with both parents	305	87.9
** **Living with mother only	38	10.9
** **Living with grandparents	4	1.2
Number of siblings aged <5 years		
** **0	150	43.2
** **1	194	55.9
** **≥2	3	0.9
Number of children in the family		
** **2-3	96	27.7
** **4–6	235	67.7
** **≥7	16	4.6
Household wealth index		
** **Lowest	67	19.3
** **Second	78	22.5
** **Middle	108	31.1
** **Fourth	58	16.7
** **Highest	36	10.4

^*^divorced, widowed, and separated.

^**^Student and house servant.

**Table 2:** Feeding practice and nutritional status of children aged 6–23 months attending growth monitoring clinic at Tsitsika Health Center, Northeast Ethiopia (*n* = 347).

Background characteristics	Frequencies	Percent (%)
Ever breast feed		
Yes	346	99.7
No	1	0.3
Current breast feeding status		
Yes	306	88.2
No	41	11.8
Introduction of complementary foods		
Early (<6 months)	71	20.5
Timely (6–8 months)	223	64.3
Late (≥9 months)	53	15.3
History of pica consumption		
Yes	87	25.0
No	254	75.0
History of cow's milk consumption		
Yes	88	25.1
No	259	74.9
Meat consumption/week		
Yes	89	25.6
No	258	74.4
Fruit consumption per week		
Yes	101	29.1
No	246	70.9
Dietary diversity score		
Poor^*^	296	85.3
Good^**^	51	14.7
Length-for-Age		
Stunted (<−2-*Z*-score)	82	23.6
Not Stunted (>−2-*Z*-score)	265	76.4
Weight-for-Length		
Wasted (<−2-*Z*-score)	54	15.6
Not Wasted (>−2-*Z*-score)	293	84.4
Weight-for-Age		
Underweight (<−2-*Z*-score)	61	17.5
Not underweight (>−2-*Z*-score)	286	82.5

Note: ^*^child received foods from <3 food groups in the previous 24 hrs.

^**^Child who received foods from ≥4 food groups in the previous 24 hrs.

**Table 3:** Morbidity and health care related characteristics of children aged 6–23 months attending growth monitoring clinic at Tsitsika Health Center, Northeast Ethiopia, 2014 (*n* = 347).

Background characteristics	Frequencies	Percent (%)
Birth place of the child		
Home	206	59.4
Health institution	141	40.6
Immunization status of the child		
Partial immunization	246	70.9
Full immunization	101	29.1
ITN^*^ utilization		
Yes	139	40.1
No	208	59.9
History of malaria infection		
Yes	50	14.4
No	297	85.6
History of intestinal parasite in the past 2 weeks		
Yes	11	3.2
No	336	96.8
History of diarrheal in the past 2 weeks		
Yes	51	14.7
No	296	85.3
History of URTIs^**^ in the past 2 weeks		
Yes	7	2.0
No	340	98.0

Note: ^*^ITN: insecticide threatened bed nets.

^**^URTIs: upper respiratory tract infections.

**Table 4:** Factors associated with anemia among children aged 6–23 months attending growth monitoring clinic at Tsitsika Health Center, Northeast Ethiopia, (*n* = 347).

Background characteristics	Anemia status of children		COR (95%: CI)	AOR (95%: CI)
	Yes	No		
Sex				
Male	131	40	**2.5 (1.57–3.95)** ^*^	**3.1 (1.60–5.81)** ^*^
Female	100	76	1.00	1.00
Age				
6–8 months	63	28	**2.1 (1.05–3.98)** ^**^	**3.5 (1.46–8.26)** ^*^
9–11 months	74	19	**3.5 (1.75–7.17)** ^*^	**9.6 (3.61–25.47)** ^*^
12–17 months	61	39	1.4 (0.75–2.69)	**2.9 (1.23–6.75)** ^**^
18–23 months	33	30	1.00	1.00
Introduction of complementary foods				
<6 months	65	6	**8.5 (3.53–20.42)** ^*^	**11.1 (4.08–30.31)** ^*^
≥9 months	41	12	**2.7 (1.34–5.37)** ^*^	**4.3 (1.78–10.18)** ^*^
6–8 months	125	98	1.00	1.00
Dietary diversity				
Poor	208	88	**2.9 (1.57–5.27)** ^*^	**3.2 (1.35–7.38)** ^*^
Good	23	28	1.00	1.00
Length-for-Age				
Stunted	70	12	**3.8 (1.95–7.29)** ^*^	**2.7 (1.20–6.05)** ^**^
Not stunted	161	104	1.00	1.00
History of diarrhoea before 2 weeks				
Yes	47	5	**5.5 (2.13–14.31)** ^*^	**4.9 (1.63–14.59)** ^*^
No	185	111	1.00	1.00
Educational level of the mother				
No formal education	109	31	**3.4 (1.947–6.083)** ^*^	**2.6 (1.26–5.27)** ^**^
Primary education	75	39	**1.9 (1.07–3.30)** ^**^	1.8 (0.83–3.71)
Secondary education and above	47	46	1.00	1.00
HH wealth index				
Lowest	53	14	**3.8 (1.57–9.12)** ^*^	**3.0 (1.01–8.88)** ^**^
Second	55	23	**2.4 (1.06–5.40)** ^**^	**2.8 (1.02–7.81)** ^**^
Middle	73	35	2.1 (0.97–4.50)	1.2 (0.46–3.28)
Fourth	32	26	1.2 (0.54–2.83)	0.9 (0.33–2.52)
Highest	18	18	1.00	1.00

Note: *P* value ∗ < 0.01, and ∗∗ = 0.01–0.05.

HH: household.
